# Redistribution of adipose tissue is associated with left atrial remodeling and dysfunction in patients with atrial fibrillation

**DOI:** 10.3389/fcvm.2022.969513

**Published:** 2022-08-11

**Authors:** Qian Chen, Xiuzhen Chen, Jiafu Wang, Junlin Zhong, Hui Zhang, Bingyuan Wu, Zhenda Zheng, Xujing Xie, Jieming Zhu, Xixiang Tang, Suhua Li

**Affiliations:** ^1^Department of Cardiovascular Medicine, The Third Affiliated Hospital, Sun Yat-sen University, Guangzhou, China; ^2^Department of Radiology, The Third Affiliated Hospital, Sun Yat-sen University, Guangzhou, China; ^3^Department of Ultrasonography, The Third Affiliated Hospital, Sun Yat-sen University, Guangzhou, China; ^4^VIP Medical Service Center, The Third Affiliated Hospital, Sun Yat-sen University, Guangzhou, China

**Keywords:** atrial fibrillation, epicardial adipose tissue, cardiac remodeling, left atrial remodeling, dysfunction

## Abstract

**Objective:**

Adipose tissue is recognized as a crucial regulator of atrial fibrillation (AF). However, the effect of epicardial adipose tissue (EAT) on the pathophysiology of AF might be different from that of other adipose tissues. The purpose of this study was to explore the distribution features of different adipose tissues in AF patients and their relationships with left atrial (LA) remodeling and function.

**Methods:**

A total of 205 participants (including 112 AF and 93 non-AF patients) were recruited. Color doppler ultrasound was used to measure the thickness of subcutaneous, extraperitoneal, and intra-abdominal adipose tissue. Cardiac CT scan was performed to measure the mean thickness of EAT surrounding the whole heart (total-EAT) and specific regions, including left atrium (LA-EAT), left ventricle, right ventricle, interventricular groove, and atrioventricular groove. LA anatomical remodeling and function were measured by echocardiography, while electrical remodeling was evaluated by P-wave duration and dispersion using Electrocardiography (obtained after cardioversion or ablation in AF patients). Relationship between the thickness of different adipose tissues and LA remodeling and function was analyzed.

**Results:**

The thickness of subcutaneous, extraperitoneal, and intra-abdominal adipose tissue was similar between AF and non-AF patients, and had no or only weak association with LA remodeling and dysfunction. However, compared to non-AF participants, total-EAT thickness significantly increased in both paroxysmal and persistent AF patients (non-AF vs. paroxysmal AF vs. persistent AF: 6.31 ± 0.63 mm vs. 6.76 ± 0.79 mm vs. 7.01 ± 1.18 mm, *P* < 0.001), which was positively correlated with the LA size and P-wave duration and dispersion, and negatively correlated with LA ejection fraction and peak strain rate. More interestingly, EAT thickness in AF patients did not increase uniformly in different regions of the heart. Compared to EAT surrounding the other regions, LA-EAT was found to accumulate more greatly, and had a closer relationship to LA remodeling and dysfunction. Multivariate logistic regression analysis also showed that LA-EAT was significantly correlated with the presence of AF (OR = 4.781; 95% CI 2.589–8.831, *P* < 0.001).

**Conclusion:**

Rather than other adipose tissues, accumulation and redistribution of EAT, especially surrounding the LA, is associated with LA remodeling and dysfunction in AF patients.

## Introduction

Atrial fibrillation (AF) is the most common arrhythmia in clinic that significantly increases the risk of stroke, heart failure, and all-cause mortality (1, 2). Although significant progress has been made in preventing and treating AF, the incidence rate continues to increase over time. Previous studies have confirmed that left atrial remodeling and dysfunction is the essential change of pathophysiology for AF (3). As the body’s largest endocrine organ, adipose tissue is considered as a crucial regulator of AF, which may affect the occurrence, progression, and prognosis of AF (4). However, due to the broad dispersion of adipose tissue throughout the body and intra-organ location, the visceral fat with high secretory activity around the heart usually has a more practical effect than fat in other parts, promoting atrial remodeling and increasing the susceptibility to AF (5). As a kind of organ-specific adipose tissue in direct contact with the surface of the heart, epicardial adipose tissue (EAT) can secrete paracrine or endocrine inflammatory cytokines, adipokines, or other factors that influence the health or diseases of the heart (6). These facts give rise to the hypothesis that adipose tissue might be redistributed in AF patients and play a role in left atrial (LA) remodeling and dysfunction. Therefore, this study aimed to explore the distribution features of different adipose tissues in AF patients and their relationships with LA remodeling and dysfunction.

## Materials and methods

### Patient characteristics

This is a single-center prospective cross-sectional study conducted in the Department of Cardiovascular Medicine, the Third Affiliated Hospital, Sun Yat-sen University. A total of 205 inpatients were enrolled between September 2020 and August 2021, including 112 patients with non-valvular AF (61 paroxysmal AF and 51 persistent AF) diagnosed according to the ESC guideline (1), and 93 patients with sinus rhythm as control group. Patients younger than 18 years old, pregnant or lactating women, patients with organic heart diseases (including valvular heart disease, hyperthyroid cardiomyopathy, dilated cardiomyopathy, and ischemic cardiomyopathy), and patients with any other systemic disorders (including hepatic disorders, renal dysfunction, neoplastic diseases, connective tissue disease, and thyroid disorders), were excluded. The research protocol has been reviewed and approved by the medical ethics committee of the Third Affiliated Hospital of Sun Yat-sen University, which is in line with the ethical guidelines of the Declaration of Helsinki in 1975 (7). All participants voluntarily participated in the study and signed informed consent. Study participants strictly followed the study protocol to reduce information bias.

### Data collection

Baseline data were collected from all subjects, including age, gender, body mass index (BMI), systolic blood pressure (SBP), diastolic blood pressure (DBP), heart rate, lifestyle (smoking and drinking), and comorbidity (hypertension, hyperlipidemia, diabetes, coronary heart disease, and stroke). Blood samples were collected after 8-h fasting and biochemical parameters were examined by Hitachi 7180 chemical analyzer, including triglyceride, total cholesterol, high-density lipoprotein cholesterol, low-density lipoprotein cholesterol, fasting plasma glucose, glycosylated hemoglobin, uric acid, blood urea nitrogen, creatinine, serum cystatin C, and estimated glomerular filtration rate (eGFR). Besides, medications that may affect the structure or function of the heart were also collected.

### Measurement of adipose tissues

Abdominal Color Doppler ultrasound was used to measure the thickness of subcutaneous, extraperitoneal, and intra-abdominal adipose tissue. Subcutaneous adipose tissue was evaluated by measuring the abdominal wall fat thickness at 1 cm above the umbilicus (longitudinal section of the line between the xiphoid process and umbilicus). Extraperitoneal adipose tissue was evaluated by measuring the distance between the left extrahepatic peritoneum and the abdominal white line under the xiphoid process. Intra-abdominal adipose tissue was evaluated by measuring the distance between the posterior wall of the abdominal aorta and peritoneum at 1 cm above the umbilicus (cross-cut between the xiphoid process and umbilicus). All adipose tissue measurements were independently performed by two experienced echocardiographers who were blinded to the clinical data of the patients. The average values were used for the statistical analysis.

Cardiac CT scan was performed to measure the thickness of EAT by using a 320 × 0.5 mm detector configuration scanner (Toshiba Aquilion One Dynamic Volume CT, Japan) with the gantry rotation time of 350 ms. Tube voltage and current were adapted to body mass index (BMI) and thoracic anatomy. Tube voltage was 100–135 kV and maximal tube current was 380–480 mA (depending on BMI and thoracic anatomy). All patients received 50–70 ml (depending on BMI) non-ionic contrast medium (Isovue-370; Bracco Diagnostics, Guangzhou, China) at a flow rate of 6.0 ml/s followed by 20 ml normal saline injected with a dual injector (Mallinckrodt Empower CTA DUAL Injector, Mallinckrodt Inc., Cincinnati, OH, United States). Bolus tracking was set in the descending aorta using a 180 Hounsfield unit threshold. We scanned with retrospective ECG-triggered dose modulation, and reconstructed at the end-diastole phase of left ventricle. The initial data set with 0.50 mm slice thickness and 0.25 mm reconstruction interval was used for further analysis. The dose-length product (DLP) was obtained from the patient protocol of the scanner. The effective dose (measured in millisieverts) was defined as the product of the DLP and a conversion coefficient for the chest. The axial source images were transferred to an image post-processing workstation (Vitrea 2.0; Vital Images, Minnetonka, MN, United States) for quantitative analysis of EAT surrounding the heart. The maximal EAT thickness was determined from the myocardial surface to the pericardium (perpendicular to the pericardium) and having threshold attenuation values of −30 to −250 Hounsfield units. The ventricular short axis view (at the basal, mid ventricular and apical levels) horizontal long axis view and short-axis view of LA with a slice thickness of 2 mm were obtained by multiplanar reconstructions of the raw data. The mean measurements of the ventricular short axis view (basal, mid, apical) were used for analyses. The short axis view of LA was reconstructed as a plane perpendicular to the long axis of standard 2- and 4-chamber views of LA at the level of the mid LA. In the ventricular short-axis view, seven segments of EAT thickness were measured, including right ventricular (RV) anterior free wall superior, RV anterior free wall inferior, RV superior wall, RV diaphragmatic wall, superior interventricular (IV) groove, inferior IV groove, and left ventricular (LV) lateral wall. In the horizontal long-axis view, four segments of EAT thickness were measured, including LV apex, RV apex, left atrioventricular (AV) groove, and right AV groove. In the short-axis view of LA, the EAT thickness was measured as the shortest distance between the mid-LA wall and three anatomical markers, including esophagus (LA-Eso), pulmonary artery (LA-PA) and descending aorta (LA-Desc Ao). Mean EAT thickness surrounding the whole heart (total-EAT) was calculated as the average value of all segments. Mean EAT thickness surrounding LA (LA-EAT) was calculated as the average value of LA-Eso, LA-PA, and LA-Desc Ao. Mean EAT thickness surrounding LV (LV-EAT) was calculated as the average value of LV apex and lateral walls. Mean EAT thickness surrounding RV (RV-EAT) was calculated as the average value of 5 segments (RV anterior free wall superior, RV anterior free wall inferior, RV superior wall, RV diaphragmatic wall, and RV apex). Mean value of EAT thickness in IV grooves (IVG-EAT) was calculated as the average value of superior and inferior IV grooves. And mean value of EAT thickness in AV groove (AVG-EAT) was calculated as the average value of the left and right AV grooves. We measured the epicardial fat by a single investigator (with 15 years’ experience in cardiac CT diagnosis and was blinded to clinical information) with digital calipers. Repeat measurements were made at intervals of more than 1 month. The intraobserver reproducibility was 0.893 [95% confidence interval (CI) 0.837–0.926]. Another investigator (with 11 years’ experience in cardiac CT diagnosis and was blinded to clinical information) selected 25 patients randomly to measure the epicardial fat. The interobserver reliability was 0.847 (95% CI 0.804–0.891).

### Evaluation of left atrial remodeling and function

Left atrial anatomical remodeling and function were evaluated by experienced registered cardiologists using Philips ie33 color Doppler ultrasound diagnostic instrument, followed the guidelines of the American Association of echocardiography (ASE) for evaluating the changes of cardiac structure and function through two-dimensional speckle tracking imaging and real-time three-dimensional imaging. The LA anteroposterior diameter (LAAPD) was measured according to the parasternal left ventricular long axis section. The left and right atrial diameter (LALRD) and LA superior and inferior diameter (LASID) were measured according to the apical four-chamber view. The mean value of LA minimum volume (LAVmin) and LA maximum volume (LAVmax) were measured according to the images of the four-chamber heart. At the same time, the LA total ejection fraction [LAtEF = (LAVmax – LAVmin)/LAVmax] and active ejection fraction [LAaEF = (LAVp – LAVmin)/LAVp] was calculated. Furthermore, the LA peak strain rate (SRs) in left ventricular systole and the peak strain rate (SRe) in early left ventricular diastole were measured by a two-dimensional speckle tracking technique.

Left atrial electrical remodeling was evaluated by measuring the P-wave duration and dispersion using 12 lead Electrocardiography during sinus rhythm. For patients with AF, sinus rhythm was achieved by cardioversion or ablation. The paper speed of ECG was set to 50 mm/s, with an amplitude of 20 mm/mv. The P-waves of select leads II, III, a VF, and V1 were identified. P-wave duration was retrieved by measuring the distance between the beginning and ending point of the P wave intersected with the equipotential line. Five continuous measurements of P-wave duration were obtained and the average value was calculated. The maximum and minimum P-wave durations were used to calculate the P-wave dispersion (Pd = maximum P-wave duration - minimum P-wave duration). All ECG recordings were analyzed independently by two investigators.

### Statistical analysis

Categorical variables were expressed as numbers and percentages, while continuous variables were expressed as mean ± standard deviation. Kolmogorov-Smirnov test was used to study the normal distribution of data. Student’s *t*-test and Mann-Whitney *U* test were used to compare continuous variables between two groups, and analysis of variance (ANOVA) or Wilcoxon signed-rank test was used to compare continuous variables among three groups. Chi-square analysis, Fisher’s Exact test, or McNemar’s test were used for proportion comparison. Pearson correlation coefficient and significance test were used to determine the association between adipose tissue and LA remodeling and dysfunction. The univariate and multivariate logistic regression models were used to analyze the correlation between EAT and the presence of AF. Model 1 was adjusted for heart rate, uric acid, and serum cystatin C, which got *P* < 0.05 in univariate analysis. Model 2 was additionally adjusted for the traditional risk factors that may affect AF, including age, gender, BMI, hypertension, hyperlipidemia, diabetes, coronary artery disease, stroke, smoking status, alcohol abuse, eGFR, and medications. *P* < 0.05 was considered statistically significant. All statistical analyses were performed using SPSS software version 20.0 (SPSS, Chicago, IL, United States).

## Results

### Patients’ characteristics

The baseline data characteristics of the study population were summarized in Table 1, including 93 patients with sinus rhythm (mean age 64.46 ± 10.92 years; 58.06% male), 61 patients with paroxysmal AF (mean age 67.34 ± 11.38 years; 65.57% male), and 51 patients with persistent AF (mean age 68.25 ± 10.40 years; 64.71% male). The mean values of heart rate (*P* < 0.001), serum uric acid (*P* = 0.042), and cystatin C (*P* = 0.001) were significantly increased in both paroxysmal and persistent AF patients when compared to those with sinus rhythm. Other baseline data did not show significant differences among groups (*P* > 0.05).

**TABLE 1:** Baseline characteristics of participants.

Variables	Sinus rhythm (*n* = 93)	Paroxysmal AF (*n* = 61)	Persistent AF (*n* = 51)	*P*-value
Age, years	64.46 ± 10.92	67.34 ± 11.38	68.25 ± 10.40	0.093
Male, n (%)	54 (58.06)	40 (65.57)	33 (64.71)	0.577
BMI, kg/m^2^	23.73 ± 2.89	24.59 ± 4.14	24.89 ± 3.37	0.106
Hypertension, n (%)	50 (53.76)	31 (50.82)	26 (50.98)	0.919
Hyperlipidemia, n (%)	36 (38.71)	20 (32.79)	19 (37.25)	0.752
DM, n (%)	24 (25.81)	20 (32.79)	20 (39.22)	0.240
CAD, n (%)	18 (19.35)	12 (20.00)	10 (19.61)	0.995
Stroke/TIA, n (%)	6 (6.45)	8 (13.11)	9 (17.65)	0.108
Current smoker, n (%)	33 (35.48)	24 (39.34)	22 (43.14)	0.658
Alcohol abuse, n (%)	13 (13.98)	8 (13.11)	6 (11.76)	0.932
SBP, mmHg	136.72 ± 19.65	135.69 ± 21.29	137.57 ± 26.28	0.902
DBP, mmHg	80.84 ± 10.81	80.13 ± 10.38	81.43 ± 13.24	0.831
HR, bpm	75.65 ± 11.61	77.75 ± 20.39	88.10 ± 15.55	<0.001
TC, mmol/L	4.43 ± 1.23	4.42 ± 1.21	4.50 ± 1.15	0.930
TG, mmol/L	1.48 ± 0.70	1.24 ± 0.63	1.44 ± 0.75	0.107
LDL-C, mmol/L	2.75 ± 1.03	2.85 ± 1.03	2.88 ± 1.01	0.716
HDL-C, mmol/L	1.05 ± 0.24	1.05 ± 0.23	1.06 ± 0.28	0.943
FBG, mmol/L	6.08 ± 1.77	6.20 ± 1.61	6.32 ± 1.53	0.701
HbA1c, %	6.47 ± 1.47	6.63 ± 1.32	6.70 ± 1.40	0.621
UA, μmol/L	394.04 ± 115.12	426.70 ± 102.12	439.08 ± 112.68	0.042
BUN, μmol/L	5.87 ± 2.49	6.01 ± 1.61	6.55 ± 2.94	0.270
SCr, μmol/L	76.11 ± 22.41	77.74 ± 17.16	84.68 ± 30.04	0.101
CysC, mg/L	0.96 ± 0.26	1.14 ± 0.31	1.15 ± 0.36	0.001
eGFR, mL/min/1.73 m^2^	81.64 ± 21.41	82.06 ± 16.62	74.36 ± 20.26	0.101
ACEI/ARB, n (%)	31 (33.33)	18 (29.51)	10 (19.61)	0.218
β-blocker, n (%)	42 (45.16)	24 (39.34)	20 (39.22)	0.698
Anticoagulant, n (%)	50 (53.76)	26 (42.62)	28 (54.90)	0.316
Statin, n (%)	69 (74.19)	36 (59.02)	32 (62.75)	0.114

The data were shown as the mean ± SD, or n (%). BMI, body mass index; DM, diabetes mellitus; CAD, coronary artery disease; TIA, transient ischemic attack; SBP, systolic blood pressure; DBP, diastolic blood pressure; HR, heart rate; TC, total cholesterol; TG, triglycerides; LDL-C, low density lipoprotein cholesterol; HDL-C, high density lipoprotein cholesterol; FBG, fasting blood glucose; UA, uric acid; BUN, blood urea nitrogen; SCr, serum creatinine; CysC, cystatin C; eGFR, estimated glomerular filtration rate; ACEI, angiotensin-converting enzyme inhibitors; ARB, angiotensin receptor blockers.

### Left atrial remodeling and dysfunction in atrial fibrillation patients

The changes in indexes of LA remodeling and dysfunction in AF patients were listed in Table 2. When compared to those in patients with sinus rhythm, variables representing LA size, including LAAPD, LALRD, LASID, LAVmax, and LAVmin, were significantly increased in patients with AF, irrespective of paroxysmal or persistent (*P* < 0.001). However, variables representing LA function, including LAtEF, LAaEF, SRs and SRe, were significantly decreased in patients with AF (*P* < 0.001). When referred to LA electrical remodeling, P-wave duration and dispersion were also significantly increased in AF patients than those in patients with sinus rhythm (*P* < 0.001). In addition, all variables of LA remodeling and dysfunction seemed to further worsen in patients with persistent AF than those with paroxysmal AF (*P* < 0.001).

**TABLE 2:** Indexes of LA remodeling and function in participants.

Variables	Sinus rhythm (*n* = 93)	Paroxysmal AF (*n* = 61)	Persistent AF (*n* = 51)	*P*-value
LAAPD, mm	29.08 ± 6.05	33.69 ± 5.60	39.14 ± 5.22	<0.001
LALRD, mm	31.29 ± 5.82	34.69 ± 6.02	37.29 ± 6.39	<0.001
LASID, mm	39.48 ± 5.78	47.67 ± 6.13	51.33 ± 6.02	<0.001
LAVmax, ml	33.15 ± 5.51	42.89 ± 9.36	49.18 ± 9.76	<0.001
LAVmin, ml	10.77 ± 3.58	18.23 ± 7.84	30.27 ± 6.96	<0.001
LAtEF,%	65.22 ± 5.59	55.81 ± 7.02	40.63 ± 6.34	<0.001
LAaEF,%	45.91 ± 5.78	41.85 ± 8.98	37.64 ± 7.57	<0.001
SRs,%	32.69 ± 9.31	19.88 ± 6.84	11.25 ± 4.82	<0.001
SRe,%	10.54 ± 2.73	7.87 ± 4.23	3.06 ± 3.38	<0.001
P-wave duration, ms	99.40 ± 6.54	107.64 ± 9.51	123.07 ± 16.09	<0.001
P-wave dispersion, ms	19.75 ± 6.28	26.87 ± 11.79	31.40 ± 15.88	<0.001

The data are shown as the mean ± SD. AF, atrial fibrillation; LAAPD, LA anteroposterior diameter; LALRD, LA left and right diameter; LASID, superior and inferior diameter of left atrium; LAVmax, LA maximum volume; LAVmin, LA minimum volume; LAtEF, LA total emptying fraction; LAaEF, LA active ejection fraction; SRs, strain rate during ventricular systole; SRe, strain rate during early ventricular diastole.

### Distribution features of adipose tissues in atrial fibrillation patients

**FIGURE 1:**
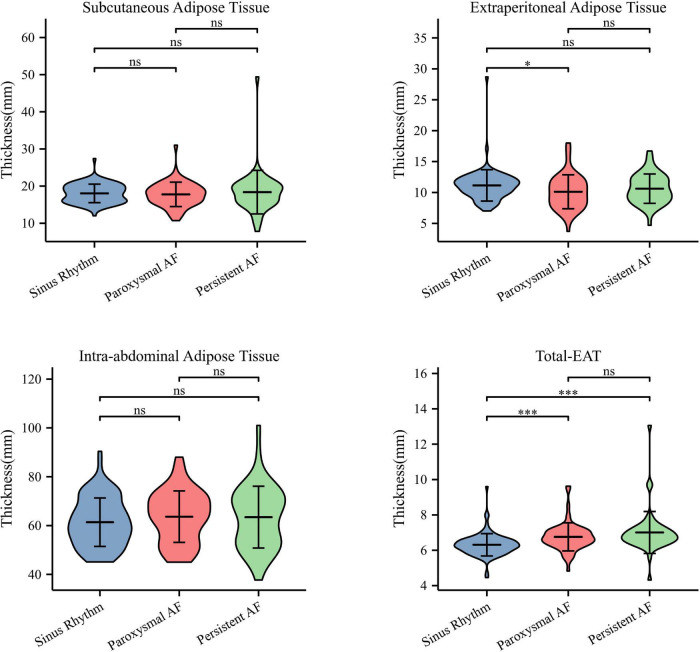
Distribution characteristics of different adipose tissues in patients with AF. Only thickness of total-EAT was significantly increased in AF patients compared to that in patients with sinus rhythm. ^∗^*P* < 0.05 and ^∗∗∗^*P* < 0.001. AF, atrial fibrillation; EAT, epicardial adipose tissue; Total-EAT, mean EAT thickness surrounding the whole heart.

As shown in Figure 1, the thicknesses of subcutaneous and intra-abdominal adipose tissues were similar between AF and non-AF patients (*P* = 0.716 and 0.382, respectively). The thickness of extraperitoneal adipose tissue tended to decrease in AF patients, although without statistical significance (*P* = 0.054). In contrast, when compared to that in non-AF patients, the thickness of total-EAT significantly increased in both paroxysmal and persistent AF patients (6.31 ± 0.63 vs. 6.76 ± 0.79 vs. 7.01 ± 1.18 mm, *P* < 0.001), indicating that accumulation of total EAT surrounding the whole heart was occurred in AF patients. Multivariate logistic regression analysis showed that total-EAT (OR = 2.615, *P* = 0.001 in Model 1; OR = 2.448, *P* = 0.005 in Model 2) was independently associated with the presence of AF (Table 3).

**TABLE 3:** Logistic regression analysis to evaluate the impact of EAT on the presence of AF.

Variables	Univariate			Model 1^a^			Model 2^b^		
	OR	95% CI	*P*-value	OR	95% CI	*P*-value	OR	95% CI	*P*-value
Total-EAT	3.321	1.920–5.746	<0.001	2.615	1.498–4.564	0.001	2.448	1.302–4.604	0.005
LA-EAT	4.737	3.126–7.180	<0.001	3.938	2.566–6.045	<0.001	4.781	2.589–8.831	<0.001
LV-EAT	1.502	1.064–2.122	0.021	1.534	0.976–2.411	0.063	1.245	0.815–1.903	0.310
RV-EAT	1.580	1.108–2.253	0.012	1.453	0.955–2.213	0.081	1.274	0.838–1.936	0.258
IVG-EAT	0.888	0.692–1.140	0.353	1.147	0.872–1.509	0.326	0.824	0.586–1.160	0.267
AVG-EAT	0.864	0.772–0.967	0.011	0.915	0.654–1.280	0.604	0.888	0.762–1.036	0.131

EAT, epicardial adipose tissue; AF, atrial fibrillation; Total-EAT, mean EAT thickness surrounding the whole heart; LA-EAT, mean EAT thickness surrounding left atrium; LV-EAT, mean EAT thickness surrounding left ventricle; RV-EAT, mean EAT thickness surrounding right ventricle; IVG-EAT, mean EAT thickness in interventricular grooves; AVG-EAT, mean EAT thickness in atrioventricular groove. ^a^ Model 1 was adjusted for HR, UA, and CysC, which have P < 0.05 in univariate regression analysis. ^b^ Model 2 was additionally adjusted for the traditional risk factors, including age, gender, BMI, hypertension, hyperlipidemia, diabetes, coronary artery disease, stroke, smoking status, alcohol abuse, eGFR, ACEI/ARB, β-blocker, anticoagulant, and statin.

**FIGURE 2:**
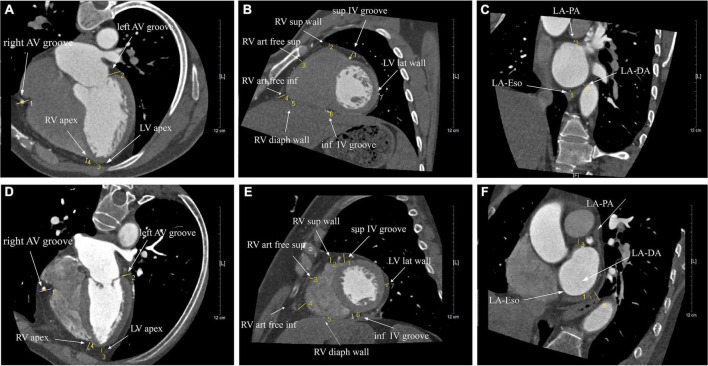
The illustration of the thickness of EAT surrounding the heart in patients with sinus rhythm **(A–C)** and AF **(E,F)**. Cardiac CT images were measured in three different views, including the parasternal short-axis view **(A,D)**, the horizontal long-axis view **(B,E)**, and the short-axis view **(C,F)**.

**FIGURE 3:**
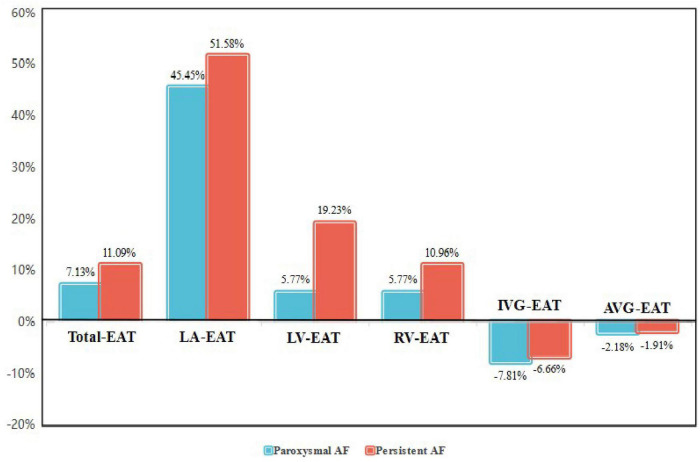
The ratio of change in EAT relative to the control group in different regions of the heart in paroxysmal and persistent AF patients. AF, atrial fibrillation; EAT, epicardial adipose tissue; Total-EAT, mean EAT thickness surrounding the whole heart; LA-EAT, mean EAT thickness surrounding left atrium; LV-EAT, mean EAT thickness surrounding left ventricle; RV-EAT, mean EAT thickness surrounding right ventricle; IVG-EAT, mean EAT thickness in interventricular grooves; AVG-EAT, mean EAT thickness in atrioventricular groove.

Interestingly, it was found that EAT in AF patients did not accumulate uniformly in different regions of the heart, and redistribution of EAT was observed. As shown in Figure 2 and Table 4, the thickness of LA-EAT (*P* < 0.001), LV-EAT (*P* = 0.007), and RV-EAT (*P* = 0.013) progressively elevated from control groups, paroxysmal AF group, to persistent AF group. However, the thickness of IVG-EAT reduced significantly in both paroxysmal and persistent AF patients (*P* = 0.031), while the thickness of AVG-EAT had no significant difference among three groups (*P* = 0.648). Furthermore, the ratio of change in EAT relative to the control group in different regions of the heart was presented in Figure 3, which indicated that the ratio of change in LA-EAT in AF patients, irrespective of paroxysmal or persistent, was much higher than those in other regions of the heart. Multivariate logistic regression analysis showed that LA-EAT (OR = 3.938; *P* < 0.001 in Model 1; OR = 4.781; *P* < 0.001 in Model 2), rather than EAT in other regions, was independently associated with the presence of AF after adjusting for other risk factors of AF (Table 3).

**TABLE 4:** Redistribution of EAT in different regions of the heart in patients with AF.

Variables	Sinus rhythm (*n* = 93)	Paroxysmal AF (*n* = 61)	Persistent AF (*n* = 51)	*P*-value
LA-EAT, mm	5.06 ± 0.80	7.36 ± 1.44	7.67 ± 2.00	0.000
LV-EAT, mm	3.12 ± 0.59	3.30 ± 1.17	3.72 ± 1.56	0.007
RV-EAT, mm	5.20 ± 0.64	5.50 ± 1.08	5.77 ± 1.72	0.013
IVG-EAT, mm	13.06 ± 2.36	12.04 ± 2.55	12.19 ± 2.94	0.031
AVG-EAT, mm	7.34 ± 0.93	7.18 ± 1.19	7.20 ± 1.35	0.648

The data are shown as the mean ± SD. AF, atrial fibrillation; EAT, epicardial adipose tissue; LA-EAT, mean EAT thickness surrounding left atrium; LV-EAT, mean EAT thickness surrounding left ventricle; RV-EAT, mean EAT thickness surrounding right ventricle; IVG-EAT, mean EAT thickness in interventricular grooves; AVG-EAT, mean EAT thickness in atrioventricular groove.

### Redistribution of adipose tissues and left atrial remodeling and dysfunction

**FIGURE 4:**
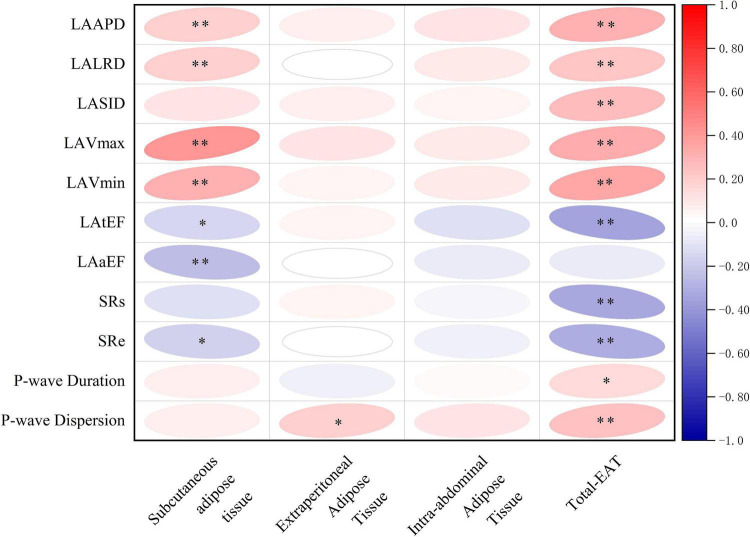
Correlations of different adipose tissues with LA remodeling and dysfunction in AF patients. ^∗^*P* < 0.05, ^∗∗^*P* < 0.01. EAT, epicardial adipose tissue; Total-EAT, mean EAT thickness surrounding the whole heart; LAAPD, LA anteroposterior diameter; LALRD, LA left and right diameter; LASID, superior and inferior diameter of left atrium; LAVmax, LA maximum volume; LAVmin, LA minimum volume; LAtEF, LA total emptying fraction; LAaEF, LA active ejection fraction; SRs, strain rate during ventricular systole; SRe, strain rate during early ventricular diastole.

As shown in Figure 4, total-EAT was significantly associated with almost all indexes of LA remodeling and dysfunction in AF patients. It seemed that total-EAT was positively correlated with LA size (including LAAPD, LALRD, LASID, LAVmax and LAVmin) and P-wave duration and dispersion, and negatively correlated with LA function (LAtEF, SRs, and SRe). Subcutaneous adipose tissue was associated with partial indexes of LA remodeling and dysfunction, including LAAPD, LALRD, LAVmax, LAVmin, LAaEF, SRs, and SRe, while extraperitoneal and intra-abdominal adipose tissues had no or only weak association with those indexes.

**FIGURE 5:**
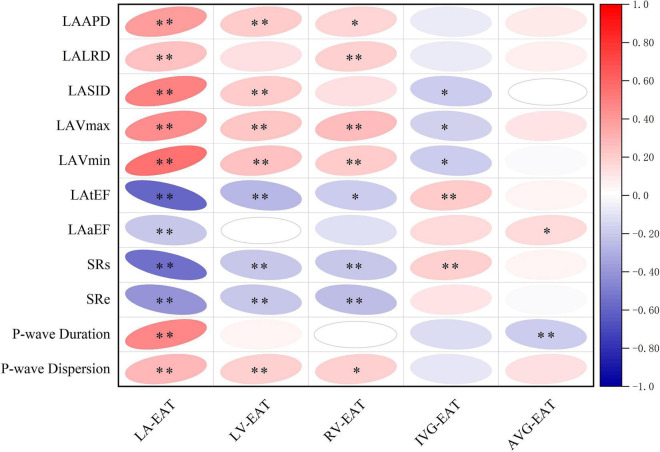
Correlation of EAT in different regions of the heart with LA remodeling and dysfunction in AF patients. ^∗^*P* < 0.05, ^∗∗^*P* < 0.01. EAT, epicardial adipose tissue; LA-EAT, mean EAT thickness surrounding left atrium; LV-EAT, mean EAT thickness surrounding left ventricle; RV-EAT, mean EAT thickness surrounding right ventricle; IVG-EAT, mean EAT thickness in interventricular grooves; AVG-EAT, mean EAT thickness in atrioventricular groove; LAAPD, LA anteroposterior diameter; LALRD, LA left and right diameter; LASID, superior and inferior diameter of left atrium; LAVmax, LA maximum volume; LAVmin, LA minimum volume; LAtEF, LA total emptying fraction; LAaEF, LA active ejection fraction; SRs, strain rate during ventricular systole; SRe, strain rate during early ventricular diastole.

**FIGURE 6:**
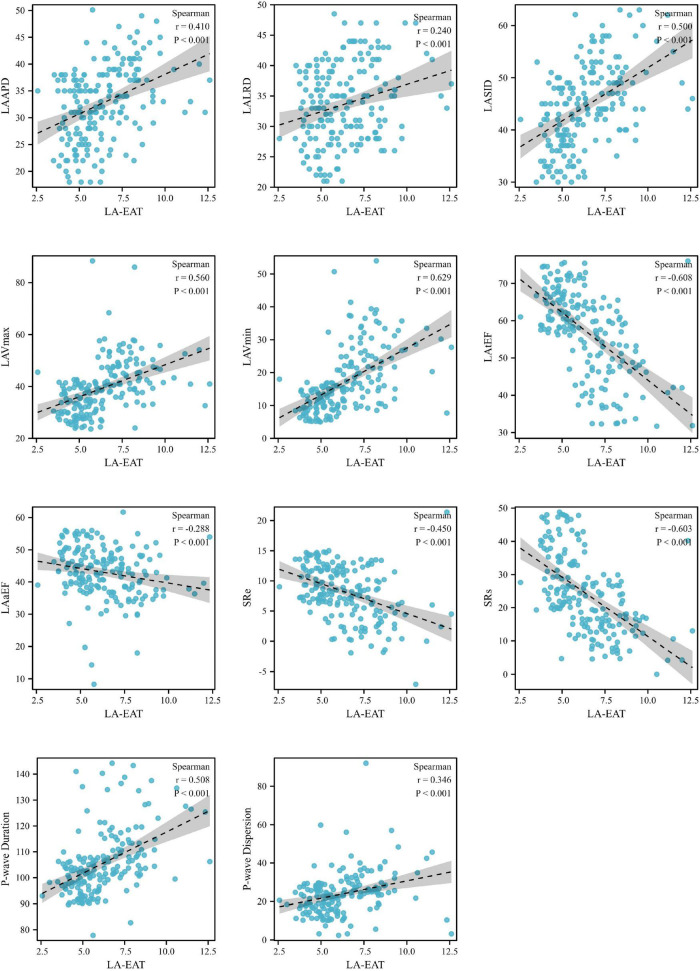
Linear trends between LA-EAT and LA indexes in AF patients. AF, atrial fibrillation; LV-EAT, mean Epicardial adipose tissue thickness surrounding left ventricle; AF, atrial fibrillation; LAAPD, LA anteroposterior diameter; LALRD, LA left and right diameter; LASID, superior and inferior diameter of left atrium; LAVmax, LA maximum volume; LAVmin, LA minimum volume; LAtEF, LA total emptying fraction; LAaEF, LA active ejection fraction; SRs, strain rate during ventricular systole; SRe, strain rate during early ventricular diastole.

When referred to EAT in different regions of the heart, as shown in Figure 5, LA-EAT was found to be significantly associated with all indexes of LA remodeling and dysfunction in AF patients (all *P* < 0.05). However, EAT in other regions were only associated with partial indexes of LA remodeling and dysfunction. Further linear trend analysis showed that LA-EAT was positively correlated with LAAPD, LALRD, LASID, LAVmax, LAVmin, and P-wave duration and dispersion (all *P* < 0.05), and negatively correlated with LAtEF, LAaEF, SRs and SRe (all *P* < 0.05) (Figure 6).

## Discussion

The issue that adipose tissue distribution affects cardiovascular disease has been widely discussed (8–10). The present study found that systemic redistribution of body adipose tissue occurred in AF patients, where the accumulation of total EAT surrounding the heart had a closer correlation with the LA remodeling and dysfunction in AF patients than the adipose tissue in other parts of the body, including subcutaneous, extraperitoneal, and intra-abdominal ones. More interestingly, when we further studied the epicardial distribution feature of adipose tissue surrounding specific regions of the heart, we found that the change in LA-EAT was particularly significant, and had a closer relationship to LA remodeling and dysfunction compared to EAT surrounding the other regions of the heart. These results suggest that systemic and epicardial distribution of adipose tissue might have an impact on the pathophysiology of AF.

Over the past decades, a growing data from epidemiological, clinical and translational studies have demonstrated that EAT is associated with the presence, severity, and recurrence of AF (8–10). Poggi et al. suggested that a tight paracrine cross-talk existed between EAT and myocardium due to their anatomical and functional features, and the dysfunctional EAT can determine a pro-inflammatory environment in the surrounding myocardial tissue (10). Batal et al. found that increased posterior LA fat thickness appears to be associated with AF burden independent of age, body mass index, or LA area (8). Despite these advances, however, significant uncertainty exists and many questions remain unanswered. The differences of the present study to previous ones are as follows: (1) Most previous studies only focused on the impact of EAT on AF, but rarely simultaneously assessed the other body fat as control. The assessment of systemic and epicardial distribution features of adipose tissues in AF patients in the present study was more detailed and comprehensive. Therefore, we make it clear that compared with the overall fat environment, changes in the local fat microenvironment of the heart have more critical value for the pathophysiology of AF. (2) For evaluating the change of LA in AF patients, traditional index of LA size or area has been used in most previous studies (11–13). Nakamori et al. confirmed the correlation between EAT and LA remodeling by measuring LA diameter and volume (13). In Yorguns’ study, EAT was associated with the presence of AF and the diameter of the LA (12). However, on the basis of traditional anatomical parameters, the present study added new functional indicators for assessing the full picture of LA remodeling and dysfunction in AF patients, including LAtEF, LAaEF, SRs, and SRe. These parameters can provide more sample and convincing evidence for our conclusion. (3) The quantitative indicators of EAT by cardiac CT scan include thickness and volume (14–16). Although EAT volume can better reflect the overall situation of adipose tissue surrounding the heart, special workstation is required, with complex measurement method and long analysis time (15). In comparison, measuring the thickness of EAT is simpler and more convenient. A recent meta-analysis found that EAT was associated with AF, measured either as volume or thickness (14). In the study, the thickness was used as the evaluation method for adipose tissue to clarify the correlation between local fat microenvironment and AF, which facilitated the follow-up clinical practice.

The latest view considers that AF is the last stage of atrial remodeling, and the main pathological mechanisms include cardiac structural and electrophysiological disorders (10). The present study found that EAT, especially LA-EAT, may contribute to cardiac structural and electrical remodeling and dysfunction, leading to the occurrence of AF. Previous studies showed that EAT can change the microenvironment and myocardial function around the LA by secreting pro-fibrosis and pro-inflammatory factors through direct contact with the LA myocardium, resulting in cardiac structural and electrical remodeling, such as myocardial fibrosis, slow conduction of electrical activities in the atrium and increased heterogeneity (17, 18). Proliferative adipose tissue can extend into the myocardial cells along with the stroma, induce atrial fibrosis through fat infiltration, and changes the connection mode between myocardial cells, resulting in voltage reduction and abnormal conduction in the LA (19–21). In addition, EAT may affect the atrial electrical activity and participate in cardiac electrophysiological remodeling by affecting the function and number of autonomic plexus around the heart, especially the function of ganglion plexus in fat pad EAT (22).

After observing that EAT is related to the occurrence, maintenance, and severity of AF, studies found that EAT can help prevent and treat AF (23–26). The association between EAT and AF is independent of systemic obesity and more substantial than that of fat in other parts of the body, suggesting the feasibility and effectiveness of EAT as a biomarker of AF in clinical settings. The relationship between LA-EAT and AF is more significant than the fat in other regions of the heart, which can provide more accurate guidance for future local research and intervention of epicardial fat. CT, MRI, and other imaging methods can accurately quantify EAT in different regions to identify the high-risk group of patients prone to AF, guide risk stratification and predict AF recurrence (27). In addition, considering the possible mechanism of EAT affecting AF, as a potential and emerging therapeutic target, EAT can be managed by reducing weight, glucose and lipid, and regulating local cardiac metabolism to improve the adverse effects of EAT on the heart (23–26). EAT can also guide radiofrequency ablation to reduce the difficulty of the operation and improve the efficiency of AF treatment by constructing the EAT model (28).

The findings of this study have to be seen in the light of some limitations. First, the sample size was relatively small. More case data is needed to improve clinical trial design, implementation, and interpretation of results. Second, this is a single-center cross-sectional study assessed the relationship between EAT and AF. Whether accumulation and redistribution of EAT is associated with the prognosis and recurrence of AF was not dynamically and regularly tracked. Therefore, a follow-up database with a broader temporal and spatial dimension must be established. Third, this study only established the relationship between EAT and LA remodeling of AF in clinical practice but did not involve mechanism research. In the near future, further basic researches to explore the pathological molecular mechanism of EAT affecting the local cardiac environment and remodeling are warranted.

To sum up, our study confirmed that EAT had a stronger correlation with AF compared with the other body adipose tissue. Accumulation and redistribution of EAT, especially surrounding the LA, is associated with LA remodeling and dysfunction in AF patients, which may be the critical link to mediating the occurrence of AF.

## Data availability statement

The original contributions presented in this study are included in the article/supplementary material, further inquiries can be directed to the corresponding authors.

## Ethics statement

The studies involving human participants were reviewed and approved by the Medical Ethics Committee of the Third Affiliated Hospital of Sun Yat-sen University. The patients/participants provided their written informed consent to participate in this study. Written informed consent was obtained from the individual(s) for the publication of any potentially identifiable images or data included in this article.

## Author contributions

SL, JMZ, and XT contributed to the study design, formal analysis, and writing – original draft. QC, JW, BW, and ZZ contributed to the data acquisition and curation. JLZ and HZ made the results visualization. QC and JW contributed to the literature research. QC, XT, and SL contributed to the writing – review and editing. All authors contributed to the article and approved the submitted version.
